# Hygiene

**Published:** 1837-04

**Authors:** 


### HYGIÈNE.

### *Case of Poisoning by Arsenic mixed with Beans.*
By MM. Barruel and Chevallier.

The following Report is valuable from the clear and methodical manner in which it is arranged.

"We, the undersigned, have been requested to analyze some French-beans, (haricots,) contained in a small flask, in order to discover whether they contain any poisonous substances, and particularly arsenic, and in what manner these substances had penetrated the beans, whether by boiling or otherwise; also to say whether two garments belonging to C. contain any portions of arsenic:—We have examined with the greatest care every part of these clothes, to discover whether there was arsenic either upon them, or in their folds, but without success. We next examined the pockets, and turned them inside out on a sheet of white paper: on examining the seams, we only found some bits of dry bread mixed with some foreign substances, and some down, but nothing had the appearance of arsenic or any other poison. Not trusting however to the appearance, we introduced the substances into a test-tube with distilled water, and boiled them for a considerable time. The liquid thus obtained had neither smell, taste, nor colour; treated by various tests, and particularly by sulphuretted hydrogen, it did not become yellow, nor furnish a precipitate. With iodine, it turned to a blue colour, indicating the presence of fecula arising from the crumbs of bread.

The beans contained in the flask were spread on white paper: we found, 1st, there were twelve in number; 2d, they were mixed with other substances, and particuarly with seeds of flax of a brown colour; thirteen of these were found. There were also a small stone, and a considerable quantity of a black substance which gave the beans a disagreeable appearance, which would have disgusted those to whom they might have been given. The little stone was broken, submitted to the action of heat on burning charcoal until it became red, but it was not altered, and gave out no smell. Six and a half of the beans were introduced into a small glass vessel, and submitted during three-quarters of an hour to the action of distilled water, assisted by heat. A part of the decoction thus obtained was filtered, and treated with sulphuretted hydrogen in the presence of the said C. Immediately the liquid became yellow, and presented all the characters of a solution of arsenious acid, treated with sulphuretted hydrogen. The same beans were several times boiled in successive portions of distilled water, in order to separate all the arsenious acid, and each decoction filtered. The filtration was very slow; it took twelve hours, and the residue was preserved. Wishing to discover if the arsenious acid had penetrated into the interior of the beans, the epidermis of five and a half was most carefully removed with a penknife; they were then introduced into the glass vessel with distilled water, and boiled. On treating the decoction with sulphuretted hydrogen, the fluid became immediately of a yellow colour, as in the former instance. These beans were boiled in successive portions of water; the decoctions were slowly filtered and the residue preserved, together with that previously obtained. The epidermis that had been removed was also boiled and filtered; the fluid was mixed with that obtained from the beans, and the residue with the two other quantities.

The experiments having proved that the beans from which the epidermis was taken contained as much arsenious acid as the others, he thought it advisable to mix together all the liquids, in order to determine the quantity of arsenious acid contained in the twelve beans. Those portions which had been already tested with sulphuretted hydrogen being mixed with the rest, a stream of sulphuretted hydrogen was passed through the liquid in order to precipitate the arsenic as a sulphuret. The liquid precipitate was poured in small quantities at a time on a very small filter, in order that the sulphuret of arsenic should not be dispersed over a large surface: this process was tedious, lasting three days, owing to the viscidity of the fluid from the fecula it contained. The sulphuret, having been collected on a small filter, was washed with distilled water, and the liquid put aside for examination. As it would have been impossible to detach the sulphuret from the filter by mechanical means, it was treated with liquor ammoniæ, so that the filter, when washed and dried, was completely colourless, proving that all was dissolved. The ammoniacal solution was then evaporated until dry, by a very gentle heat, in a porcelain capsule. As fast as the ammonia evaporated, zones of a yellow colour were seen adhering to the sides of the capsule. The residuum, which was of a yellow colour, was dissolved in a quantity of a solution of caustic potash sufficient to detach it completely from the sides of the capsule to which it strongly adhered: this solution was evaporated in a watch-glass after having been mixed with a certain quantity of black flax, and, when sufficiently dried, the watch-glass was broken, and all was introduced into an apparatus, in which it was subjected to distillation. The sulphuret of arsenic being decomposed in this operation, the metallic arsenic was condensed, and weighed one centigramme, which is equal to thirteen millegrammes of arsenious acid.

As we were also required to ascertain if the beans contained any other poisonous substance, the liquors from which the sulphuret of arsenic had been obtained by filtration were evaporated in a porcelain capsule by means of a gentle heat, to the consistence of extract. This extract produced slight acid reaction with litmus paper: it had neither a bitter nor an acid taste: we triturated it during two hours with successive quantities of alcohol at 36°: the alcohol became of a slightly yellow colour, and when evaporated to dryness it left a hardly appreciable residuum of a yellow colour and slightly salt taste; when treated with concentrated nitric acid, it changed to no colour indicating the presence of a vegetable poison. This negative result was foreseen, as the alcoholic extract had no other taste than that of common salt. That part of the extract insoluble in alcohol was treated with water, which almost wholly dissolved it, except some flakes of vegetable matter. The aqueous solution, when filtered, was not changed by the addition of hydrosulphate of ammonia; the tincture of iodine gave a blue precipitate from the fecula of the beans.

The solid residuum formed, 1, of the beans treated with their epidermis, 2, of the beans without their epidermis, 3, of the epidermis which had been removed, was introduced into a flask and soaked for twenty-four hours in water saturated with sulphuretted hydrogen. This residuum did not change colour, indicating that it no longer contained arsenious acid; and also that no metallic oxides capable of being coloured by sulphuretted hydrogen were present. The residuum separated from the fluid was treated several times with boiling alcohol. The alcoholic solutions, evaporated to dryness, left a certain quantity of fatty matter in which no trace of an organic poison could be detected. Finally, the residuum of the alcoholic solution was again dried, then carbonized and incinerated in a new porcelain crucible; the result was a large cinder, in which the presence of carbonate and phosphate of lime, silica, and a small quantity of oxide of iron, without any other mineral, was demonstrated. From these facts it is proved,

1. That the beans we examined contained arsenious acid (white arsenic).
2. That the arsenious acid appeared to have penetrated the beans by ebullition, the acid having been recognized in the beans deprived of their epidermis.
3. That the proportion of the poison was thirteen milligrammes to twelve beans.
4. That the beans contained no other poison.
5. That the clothes and pockets contained no poison.

*Annales d'Hygiène publique et de Médecine Légale, Avril,* 1836.

